# Supplementary material for: The evolution of host-symbiont dependence
Source: Nat Commun. 2017 Jul 4;8:15973. doi: 10.1038/ncomms15973 (PMC5500886; doi:10.1038/ncomms15973)
Supplement: Supplementary Information [file ncomms15973-s1.pdf]

Title of file for HTML: Supplementary Information
Description: Supplementary Figures, Supplementary Tables and Supplementary References

Title of file for HTML: Supplementary Data 1
Description: Supplementary R code

Title of file for HTML: Peer Review File
Description:

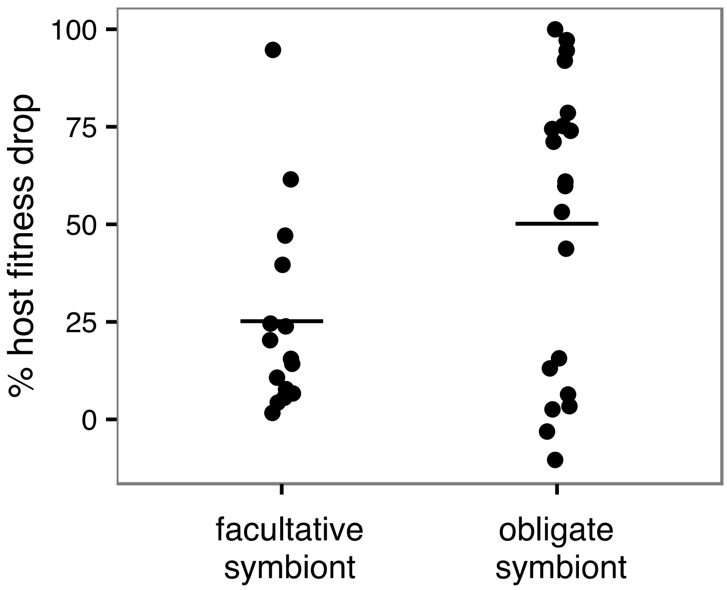

Supplementary Figure 1. Obligate symbionts lead to a higher reduction in host fitness when removed. Each data point represents one host-symbiont combination and the mean % host fitness reduction for symbionts that are facultative and obligate are shown as horizontal bars (facultative symbiont = 25.19 and obligate symbiont = 47.49). Mean host fitness reduction was higher for hosts with obligate symbionts (symbiont phylogeny: BPMM, posterior mode = -23.95, CI = -48.36, 10.64, pMCMC = 0.02). $N_{symbioses}$ = 35 and $N_{datapoints}$ = 284.

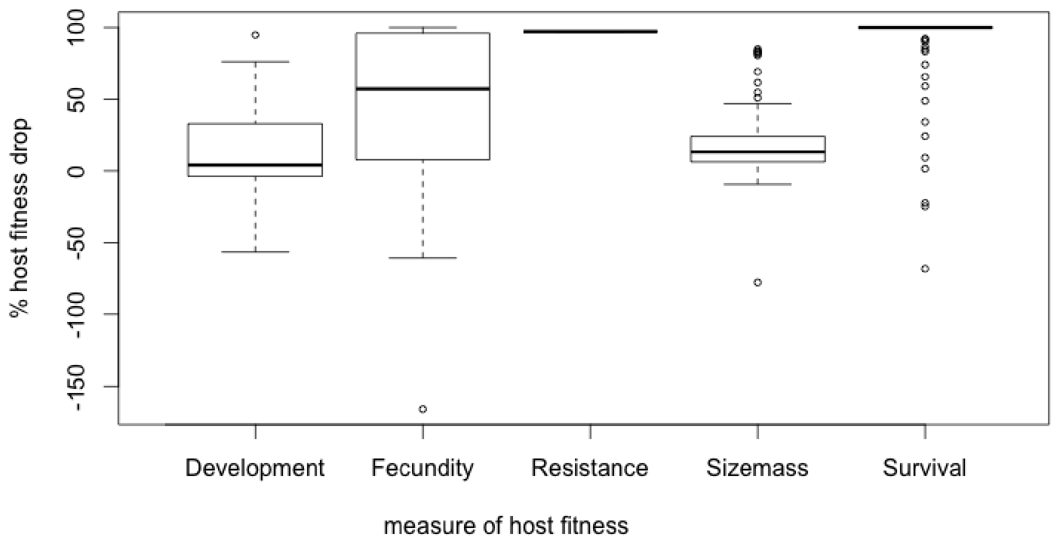

Supplementary Figure 2. No differences in host fitness drop between different methods of measuring host fitness. Mean % host fitness drop for each of the 5 categories of how host fitness was measured. In our sensitivity analyses, we removed the category 'resistance' due to the fact we only had one observation of host fitness measured in this way. We found no overall differences in % host fitness drop between different methods of measuring host fitness, when controlling for both symbiont and host phylogeny (Supplementary Tables 8 and 9). Bold horizontal bars show the median value for each treatment, boxes show the first and third quartiles and whiskers show the minimum and maximum values.

Supplementary Table 1. Summary of full dataset. Raw means are presented for % host fitness drop and symbiont genome size for each level of each fixed effect.

		**Mean host fitness drop**	**Mean symbiont genome size (Mb)**
**Transmission mode**	Horizontal	21.18	7.09
	Vertical	51.78	1.52
**Symbiont function**	Nutritional	42.71	2.12
	Defensive	28.25	2.49
**Intra/extracellular**	Intracellular	38.56	2.13
	Extracellular	56.06	3.45

Supplementary Table 2. Analysis of the effect of transmission mode, intra/extracellular and symbiont function on fitness drop of host on symbiont removal and symbiont genome size, taking into account symbiont phylogenetic relationships using MCMCglmm. $N_{symbioses} = 106$, $N_{symbiont\ species} = 58$, $N_{studies} = 84$, $N_{datapoints} = 378$.

Response: Fitness drop

**Fixed Effect**		**N**	**Posterior mode (CI)**	**pMCMC**
Transmission mode	Horizontal	125	14.33 (-32.64, 57.09)	
	Vertical	253	46.65 (13.66, 84.20)	
	Difference		-36.58 (-67.54, -8.45)	**0.01**
Intra/extracellular	Intracellular	269	-15.05 (-56.12, 32.29)	
	Extracellular	109	12.55 (-40.43, 50.96)	
	Difference		19.10 (-3.88, 49.16)	**0.07**
Function	Defensive	27	7.28 (-41.41, 50.63)	
	Nutritional	207	41.98 (11.60, 67.08)	
	Unknown	144	24.98 (-12.63, 68.87)	
	Difference (Defensive & Nutritional)		-28.75 (-66.80, 2.79)	**0.03**

Response: Genome size

**Fixed Effect**		**N**	**Posterior mode**	**pMCMC**
Transmission mode	Horizontal	125	8.46 (5.87, 9.96)	
	Vertical	253	2.77 (0.83, 4.41)	
	Difference		-5.56 (-6.74, -4.10)	**<0.001**
Intra/extracellular	Intracellular	269	6.27 (4.68, 8.85)	
	Extracellular	109	8.44 (6.04, 9.93)	
	Difference		1.34 (0.06, 2.85)	**0.03**
Function	Defensive	27	8.07 (5.84, 9.93)	
	Nutritional	207	7.74 (6.44, 9.85)	
	Unknown	144	8.79 (7.29, 11.38)	
	Difference (Defensive & Nutritional)		-0.08 (-1.47, 1.65)	**0.52**

Correlations

Fitness drop : Genome size	Phylogenetic correlation	0.02 (-16.23, 3.75)	**0.38**
	Phenotypic correlation	0.03 (-0.20, 0.20)	**0.51**

Supplementary Table 3. Analysis of the effect of transmission mode, intra/extracellular and symbiont function on fitness drop of host on symbiont removal and symbiont genome size, taking into account host phylogenetic relationships using MCMCglmm. $N_{symbioses}$ = 106, $N_{host\ species}$ = 89, $N_{studies}$=84, $N_{datapoints}$ = 378.

Response: Fitness drop

**Fixed Effect**		**N**	**Posterior mode (CI)**	**pMCMC**
Transmission mode	Horizontal	125	6.98 (-40.04, 40.73)	
	Vertical	253	44.13 (6.58, 77.69)	
	Difference		-39.33 (-67.96, -9.13)	**0.003**
Intra/extracellular	Intracellular	269	-20.30 (-54.07, 21.18)	
	Extracellular	109	0.48 (-32.24, 46.09)	
	Difference		20.16 (-6.31, 44.57)	**0.06**
Function	Defensive	27	5.18 (-37.09, 42.21)	
	Nutritional	207	38.28 (9.70, 62.06)	
	Unknown	144	23.57 (-10.76, 62.47)	
	Difference (Defensive & Nutritional)		-24.93 (-63.44, -4.84)	**0.01**

Response: Genome size

**Fixed Effect**		**N**	**Posterior mode**	**pMCMC**
Transmission mode	Horizontal	125	7.97 (5.72, 9.27)	
	Vertical	253	3.41 (1.94, 4.97)	
	Difference		4.21 (3.09, 5.56)	**<0.001**
Intra/extracellular	Intracellular	269	6.30 (4.46, 7.96)	
	Extracellular	109	7.78 (6.07, 9.61)	
	Difference		-0.0001 (0.27, 2.74)	**0.005**
Function	Defensive	27	8.14 (6.02, 9.58)	
	Nutritional	207	7.17 (5.59, 9.08)	
	Unknown	144	8.07 (6.50, 10.33)	
	Difference (Defensive & Nutritional)		0.52 (-0,64, 1.60)	**0.19**

Correlations

Fitness drop : Genome size	Phylogenetic correlation	-0.62 (-0.84, 0.89)	**0.49**
	Phenotypic correlation	-0.01 (-0.21, 0.19)	**0.53**

Supplementary Table 4. Analysis of the effect of transmission mode, intra/extracellular and symbiont function on fitness drop of host on symbiont removal and symbiont genome size, taking into account symbiont phylogenetic relationships using MCMCglmm. $N_{symbioses}$ = 106, $N_{symbiont\ species}$ = 58, $N_{studies}$= 84, $N_{datapoints}$ = 378, with transmission specific correlations between genome size and host fitness drop.

Response: Fitness drop

**Fixed Effect**		**N**	**Posterior mode (CI)**	**pMCMC**
Transmission mode	Horizontal	125	0.14 (-36.70, 51.90)	
	Vertical	253	55.52 (10.88, 92.76)	
	Difference		-41.16 (-75.87, -8.72)	**0.007**
Intra/extracellular	Intracellular	269	-5.29 (-55.99, 26.93)	
	Extracellular	109	7.47 (-33.77, 51.33)	
	Difference		17.60 (-7.24, 46.55)	**0.08**
Function	Defensive	27	6.62 (-39.82, 48.97)	
	Nutritional	207	42.38 (11.57, 68.79)	
	Unknown	144	19.42 (-13.28, 70.13)	
	Difference		-36.54 (-68.65, 4.93)	**0.05**

Response: Genome size

**Fixed Effect**		**N**	**Posterior mode (CI)**	**pMCMC**
Transmission mode	Horizontal	125	8.15 (5.23, 12.19)	
	Vertical	253	3.35 (1.56, 4.65)	
	Difference		4.63 (1.86, 8.68)	**0.008**
Intra/extracellular	Intracellular	269	6.43 (3.10, 10.20)	
	Extracellular	109	8.52 (5.08, 12.43)	
	Difference		1.92 (0.71, 3.12)	**0.003**
Function	Defensive	27	8.48 (5.01, 12.17)	
	Nutritional	207	7.12 (4.35, 11.16)	
	Unknown	144	8.80 (5.56, 12.45)	
	Difference (Defensive & Nutritional)		0.46 (-0.73, 1.76)	**0.19**

Transmission specific correlations

Fitness drop : Genome size	Horizontal	1.03 (-31.51, 61.49)	
	Vertical	-0.14 (-30.54, 6.65)	
	Difference	1.17 (-0.98, 54.85)	**0.25**

Supplementary Table 5. Analysis of the effect of transmission mode, intra/extracellular and symbiont function on fitness drop of host on symbiont removal and symbiont genome size, taking into account host phylogenetic relationships using MCMCglmm. $N_{symbioses}$ = 106, $N_{symbiont\ species}$ = 89, $N_{studies}$ = 84, $N_{datapoints}$ = 378, with transmission specific correlations between genome size and host fitness drop.

Response: Fitness drop

**Fixed Effect**		**N**	**Posterior mode (CI)**	**pMCMC**
Transmission mode	Horizontal	125	0.06 (-32.27, 40.46)	
	Vertical	258	37.39 (9.12, 78.69)	
	Difference		-32.75 (-77.23, -7.87)	**0.03**
Intra/extracellular	Intracellular	274	-10.12 (-52.38, 22.97)	
	Extracellular	109	4.91 (-33.16, 50.38)	
	Difference		16.65 (-4.96, 46.05)	**0.08**
Function	Defensive	27	-0.93 (-40.38, 39.37)	
	Nutritional	212	33.30 (10.67, 64.44)	
	Unknown	144	30.79 (-16.18, 58.46)	
	Difference (Defensive & Nutritional)		-38.28 (-62.70, -7.37)	**0.01**

Response: Genome size

**Fixed Effect**		**N**	**Posterior mode (CI)**	**pMCMC**
Transmission mode	Horizontal	125	7.95 (3.52, 13.01)	
	Vertical	258	3.95 (2.44, 4.88)	
	Difference		4.07 (-0.51, 8.78)	**0.04**
Intra/extracellular	Intracellular	274	5.65 (2.24, 10.20)	
	Extracellular	109	7.84 (4.07, 11.84)	
	Difference		1.31 (0.07, 2.58)	**<0.001**
Function	Defensive	27	8.24 (4.19, 12.20)	
	Nutritional	212	7.29 (2.97, 10.96)	
	Unknown	144	8.74 (4.85, 12.75)	
	Difference (Defensive & Nutritional)		0.98 (0.44, 1.53)	**<0.001**

Transmission specific correlations

Fitness drop : Genome size	Horizontal	0.15 (-38.88, 49.94)	
	Vertical	-0.11 (-23.30, 8.47)	
	Difference	0.72 (-42.59, 58.77)	**0.37**

Supplementary Table 6. Analysis of the effect of transmission mode and age of symbiosis on fitness drop of host on symbiont removal and symbiont genome size, taking into account symbiont phylogenetic relationships using MCMCglmm. This analysis uses a subset of the dataset where we have data on age of symbiosis: $N_{symbioses} = 54$, $N_{symbiont\ species} = 26$, $N_{studies} = 43$, $N_{datapoints} = 100$.

Response: Fitness drop

**Fixed Effect**		**N**	**Posterior mode (CI)**	**pMCMC**
Transmission mode	Horizontal	35	33.51 (-11.41, 69.93)	
	Vertical	65	59.30 (25.54, 116.02)	
	Difference		-44.81 (-87.84, -5.41)	**0.02**
Age	Difference of slope from 0		0.14 (-0.27, 0.47)	**0.3**

Response: Genome size

**Fixed Effect**		**N**	**Posterior mode (CI)**	**pMCMC**
Transmission mode	Horizontal	35	7.82 (1.39, 12.57)	
	Vertical	65	2.51 (0.58, 4.58)	
	Difference		4.16 (-0.58, 11.21)	**0.04**
Age	Difference of slope from 0		-0.01 (-0.02, 0.004)	**0.1**

Supplementary Table 7. Analysis of the effect of transmission mode and age of symbiosis on fitness drop of host on symbiont removal and symbiont genome size, taking into account host phylogenetic relationships using MCMCglmm. This analysis uses a subset of the dataset where we have data on age of symbiosis: $N_{symbioses}$ = 54, $N_{symbiont\ species}$ = 53, $N_{studies}$ = 43, $N_{datapoints}$ = 100.

Response: Fitness drop

**Fixed Effect**		**N**	**Posterior mode (CI)**	**pMCMC**
Transmission mode	Horizontal	35	31.98 (-14.37, 80.06)	
	Vertical	65	77.84 (26.36, 124.39)	
	Difference		-40.25 (-94.28, 6.46)	**0.04**
Age	Difference of slope from 0		-0.0007 (-0.50, 0.36)	**0.43**

Response: Genome size

**Fixed Effect**		**N**	**Posterior mode (CI)**	**pMCMC**
Transmission mode	Horizontal	35	6.10 (-2.66, 15.94)	
	Vertical	65	3.05 (0.78, 4.77)	
	Difference		4.51 (-5.42, 13.12)	**0.13**
Age	Difference of slope from 0		-0.003 (-0.01, 0.006)	**0.28**

Supplementary Table 8. Analysis of the effect of the way in which host fitness was measured on fitness drop of host, taking into account symbiont phylogenetic relationships using MCMCglmm. The category 'resistance' has been removed for this analysis due to lack of replication. $N_{symbioses}$ = 38, $N_{symbiontspecies}$ = 27, $N_{studies}$ = 37, $N_{datapoints}$ = 300.

**Response: Fitness drop**				
**Fixed Effect**		**N**	**Posterior mode (CI)**	**pMCMC**
**Host fitness measure**	Development	16	36.58 (14.57, 68.14)	
	Fecundity	97	47.19 (30.97, 80.69)	
	Size/Mass	107	45.02 (21.92, 74.47)	
	Survival	80	52.32 (23.12, 80.59)	
**Differences**	Diff between development time & fecundity		-18.86 (-29.64, -2.88)	**0.01**
	Diff between development time & size/mass		-8.91 (-22.84, 7.69)	**0.18**
	Diff between development time & survival		-10.17 (-30.01, 6.49)	**0.09**
	Diff between fecundity & size/mass		9.44 (-0.09, 18.90)	**0.31**
	Diff between fecundity & survival		3.02 (-14.33, 17.81)	**0.36**
	Diff between size/mass & survival		-8.77 (-24.22, 11.13)	**0.25**

Supplementary Table 9. Analysis of the effect of the way in which host fitness was measured on fitness drop of host, taking into account host phylogenetic relationships using MCMCglmm. The categories 'resistance' has been removed for this analysis due to lack of replication. $N_{hostspecies}$ = 31, $N_{studies}$ = 40, $N_{datapoints}$ = 304.

**Response: Fitness drop**				
**Fixed Effect**		**N**	**Posterior mode (CI)**	**pMCMC**
**Host fitness measure**	Development	16	32.54 (5.14, 59.86)	
	Fecundity	99	48.76 (26.97, 79.06)	
	Size/Mass	107	37.12 (14.59, 66.13)	
	Survival	82	40.26 (10.34, 66.31)	
**Differences**	Diff between development time & fecundity		-22.23 (-32.77, -5.41)	**0.004**
	Diff between development time & size/mass		-6.51 (-25.28, 4.74)	**0.12**
	Diff between development time & survival		-9.96 (-25.59, 7.85)	**0.18**
	Diff between fecundity & size/mass		11.71 (0.54, 18.55)	**0.02**
	Diff between fecundity & survival		9.45 (-3.51, 24.04)	**0.07**
	Diff between size/mass & survival		0.76 (-14.67, 18.05)	

Supplementary Table 10. Analysis of the effect of transmission mode on fitness drop of host on symbiont removal and symbiont genome size, taking into account symbiont phylogenetic relationships using MCMCglmm. This analysis uses a subset of the dataset where host fitness was measured as fecundity: $N_{symbioses} = 26$, $N_{symbiontspecies} = 18$, $N_{studies} = 29$, $N_{datapoints} = 102$.

**Response: Fitness drop**				
**Fixed Effect**		**N**	**Posterior mode (CI)**	**pMCMC**
Transmission mode	Horizontal	21	-15.96 (-82.24, 41.79)	
	Vertical	81	23.09 (-30.49, 84.30)	
	Difference		-50.89 (-85.68, -4.41)	**0.02**
**Response: Genome size**				
**Fixed Effect**		**N**	**Posterior mode**	**pMCMC**
Transmission mode	Horizontal	21	8.61 (6.68, 10.62)	
	Vertical	81	1.59 (0.36, 3.15)	
	Difference		7.02 (5.16, 8.47)	**<0.001**
**Correlations**				
Fitness drop : Genome size	Phylogenetic correlation		-0.73 (-0.95, 0.70)	**0.34**
	Phenotypic correlation		-0.07 (-0.31, 0.29)	**0.48**

Supplementary Table 11. Analysis of the effect of transmission mode on fitness drop of host on symbiont removal and symbiont genome size, taking into account host phylogenetic relationships using MCMCglmm. This analysis uses a subset of the dataset where host fitness was measured as fecundity: $N_{hostspecies}$ = 22, $N_{studies}$ = 30, $N_{datapoints}$ = 102.

**Response: Fitness drop**				
**Fixed Effect**		**N**	**Posterior mode (CI)**	**pMCMC**
Transmission mode	Horizontal	21	-34.20 (-84.73, 29.34)	
	Vertical	81	23.11 (-28.64, 72.20)	
	Difference		-43.32 (-86.98, -12.12)	**0.006**
**Response: Genome size**				
**Fixed Effect**		**N**	**Posterior mode**	**pMCMC**
Transmission mode	Horizontal	21	9.01 (6.37, 10.82)	
	Vertical	81	1.96 (0.03, 3.49)	
	Difference		7.37 (4.39, 9.68)	**<0.001**

**Correlations**			
Fitness drop : Genome size	Phylogenetic correlation	-0.52 (-0.89, 0.86)	**0.50**
	Phenotypic correlation	-0.02 (-0.30, 0.33)	**0.49**

Supplementary Table 12. Analysis of the effect of transmission mode, intra/extracellular and symbiont function on fitness drop of host on symbiont removal and symbiont genome size, taking into account symbiont phylogenetic relationships using MCMCglmm. Two symbiont species, *Verminephrobacter* and *Regiella insecticola* have been reassigned as horizontal transmission, to account for the possible influence of biparental transmission on host fitness drop. $N_{symbioses}$ = 106, $N_{symbiont\ species}$ = 58, $N_{studies}$ = 84, $N_{datapoints}$ = 378.

Response: Fitness drop

**Fixed Effect**		**N**	**Posterior mode (CI)**	**pMCMC**
Transmission mode	Horizontal	133	30.16 (-9.14, 62.49)	
	Vertical	245	58.56 (19.80, 97.33)	
	Difference		-26.64 (-53.44, -6.61)	**0.002**
Intra/extracellular	Intracellular	269	6.67 (-30.05, 37.90)	
	Extracellular	109	33.46 (-9.61, 62.70)	
	Difference		23.43 (-8.51, 43.74)	**0.07**
Function	Defensive	27	26.27 (-11.34, 59.76)	
	Nutritional	207	44.00 (17.67, 72.20)	
	Unknown	144	39.07 (-0.80, 70.95)	
	Difference (Defensive & Nutritional)		-27.27 (-49.91, 10.38)	**0.12**

Response: Genome size

**Fixed Effect**		**N**	**Posterior mode**	**pMCMC**
Transmission mode	Horizontal	133	5.62 (3.75, 7.81)	
	Vertical	245	1.19 (-0.51, 3.63)	
	Difference		4.23 (2.95, 5.72)	**<0.001**
Intra/extracellular	Intracellular	269	4.69 (2.52, 7.04)	
	Extracellular	109	5.91 (3.61, 8.01)	
	Difference		0.98 (-0.43, 2.64)	**0.07**
Function	Defensive	27	6.04 (3.63, 7.96)	
	Nutritional	207	6.37 (5.01, 8.89)	
	Unknown	144	7.61 (5.68, 9.78)	
	Difference (Defensive & Nutritional)		-0.94 (-3.02, 0.51)	**0.12**

Correlations

Fitness drop : Genome size	Phylogenetic correlation	-0.89 (-0.96, 0.84)	**0.40**
	Phenotypic correlation	-0.05 (-0.18, 0.20)	**0.48**

Supplementary Table 13. Summary of host fitness data. Studies from which we used data on mean % host fitness drop and the sample size (number of measures of host fitness) for each symbiont species. $N_{datapoints}$ is the number of host fitness measures found for removal of each symbiont species.

**Symbiont**	**Mean % host fitness drop**	**Study Reference**	**Details**	$N_{datapoints}$
Actinobacteria	87.03	[17]	Means and fitness change from Fig. 3 for aposymbiotic and control individuals	2
Azospirilllum	12.28	[18]	Means taken from Tables 1, 2c, 3, 4, 5	
Azospirilllum	5.57	[3]	Means taken from Tables 1 & 2	70
Azospirilllum	4.28	[4]	Means taken from Table 3 (0 N fertilizer) & Table 4 (0 N fertilizer)	
Blochmannia floridanus	70.56	[5]	Means taken from text (page 4, Fig. 4)	2
Blochmannia floridanus	86.50	[6]	Means taken from text (page 3, Fig. 1)	
Bradyrhizobium	76.97	[7]	Means taken from Table 1 (local strains), only shoot dry weight	
Bradyrhizobium	36.86	[8]	Means taken from Table 5, seed yield, no phosphorus treatment	28
Bradyrhizobium	20.93	[9]	Means taken from Table 2, grain yield (0 N fertilizer)	
Buchnera	71.13	[10]	Means calculated from Fig. 1b, took end point	7
Buchnera	75.26	[11]	Means taken from Table 1 & 2	
Burkholderia	20.28	[12]	Means calculated from Fig. 3 C, D, E, F, both male and female	8
Coriobacterium glomerans	59.02	[13]	Means calculated from Fig. 1	1
Coxiella	59.75	[14]	Means taken from Table 1	7
Erwinia	23.81	[15]	Means taken from Table 1	
Erwinia dacicola	43.80	[16]	Means taken from text, page 4	10
Frankia	61.50	[17]	Means taken from Table 2, total plant dry weight only *Frankia*	1
Glomeribacter gigasporarum	53.17	[18]	Means calculated from Fig. 8 & Fig. 10, only end points taken	2
Gordonibacter	6.58	[13]	Means calculated from Fig. 1	1
Hamiltonella	13.10	[19]	Means taken from Table 2 &	13

defensa			Fig. 1a	
Hamiltonella defensa	6.40	20	Means calculated from raw data given in Appendix 1	
Hamiltonella defensa	-10.45	21	Means calculated from Fig. 1A for strains Co08, Co23, Co26 & Co37	
Nardonella	15.68	22	Means calculated from Fig. 3, both male and female	5
symbiont of *Plautia stali*	99.08	23	Raw data provided by Takahiro Hosokawa	70
Regiella insecticola	-3.77	24	Means calculated from Fig. 1a	
Regiella insecticola	-2.47	19	Means taken from Table 2 & Fig. 1a	6
Regiella insecticola	34.15	25	Calculated means from raw data from Fig. 2 provided by Christoph Vorburger	
Rhodococcus rhodnii	7.73	26	Means taken from Table 1 sym *in vitro* and apo *in vitro* only for unfed individuals	1
Rickettsia	-26.17	27	Means taken from Table 1, excluding individuals infected with both symbionts.	
Rickettsia	-10.78	28	Means calculated from Fig. 1 and Table 1	
Rickettsia	-8.79	29	Means taken from Table 1 Means taken from Table 1	29
Rickettsia	0.93	30	Means taken from Table 1	
Rickettsia	33.60	31	Calculated means of Fig. 3 from raw data provided by Martha Hunter	
Serratia symbiotica	-23.67	27	Means taken from Table 1, excluding individuals	
Serratia symbiotica	81	28	Means calculated from Fig. 1 and Table 1	
Serratia symbiotica	15.07	32	Means calculated from Fig. 1	31
Serratia symbiotica	6.54	24	Means calculated from Fig. 1a	
Serratia symbiotica	23.21	19	Means taken from Table 2 & Fig. 1a	
Serratia symbiotica	-7.83	33	Means taken from Table 1	
Sinorhizobium meliloti	14.26	34	Means taken from Fig. 1 &2	4
Spirochaetes bacteria	94.73	35	Means taken from Table 1, only when B and no S, F or P	1
Spiroplasma	-22.45	36	Means taken from Table 4	
Spiroplasma	37.72	37	Means calculated from Table 1 & Fig. 1	10
Streptomyces	91.96	38	Mean calculated from text page 3	1
Verminephrobacter	31.05	39	Means calculated from Fig. 4 (only hatched data)	2
Wigglesworthia glossinidius	74	40	Means calculated from Fig. 5, only took end point	10

Wolbachia	-1.98	[41]	Means taken from text page 5	
Wolbachia	-4.75	[42]	Means taken from Table 3	
Wolbachia	100	[43]	Means taken from Table 3 only from tetracycline	
Wolbachia	60.94	[44]	Means calculated from Fig 1., highest antibiotic concentration	
Wolbachia	17.83	[45]	Means taken from Table 1, only WxW and UxU	52
Wolbachia	94.24	[46]	Means taken from Table 2	
Wolbachia	-12.52	[47]	Means taken from Table 1	
Wolbachia	6.75	[48]	Means calculated from Fig. 2, 3 & 4	
Wolbachia	14.90	[49]	Means taken from Fig. 1 legend	
Wolbachia	74.41	[50]	Means calculated from Fig. 3	
chemoautotrophic symbiont of *Codakia*	47.06	[51]	Means taken from text on page 7	1

Supplementary Table 14. Summary of symbiont transmission modes, genome sizes and age of association with hosts.

**Symbiont species**	**Transmission mode**	**General reference(s)**	**Symbiont genome size**	**Age of symbiosis in millions of years (mean)**	**Genome/age reference(s)**
Actinobacteria	Vertical	[1,52]	-	-	-
Aliivibrio fischeri	Horizontal	[53]	4.27	60	Ned Ruby, pers.comm.
Nostoc azollae (Anabaena azollae)	Vertical	[54,55]	5.49	140	[54]
Azobacteroides pseudotrichonymphae	Vertical	[56]	1.11	-	-
Azospirilllum	Horizontal	-	7.39	-	-
Baumannia cicadellinicola	Vertical	[57]	0.68	70-100 (85)	[58]
Blattabacterium	Vertical	[57]	0.64	150	[59]
Blochmannia floridanus	Vertical	[57]	0.71	50	[60]
Bradyrhizobium	Horizontal	-	9.21	62	[61]
Buchnera	Vertical	[62]	0.64	180	[57]
Burkholderia	Horizontal	[12]	6.96	-	-
Burkholderia brachyanthoides	Vertical	[63]	3.55	12	[64]
Burkholderia calva	Vertical	[63]	4.21	12	[64]
Burkholderia crenata	Vertical	[63]	2.84	12	[64]
Burkholderia humilis	Vertical	[63]	5.15	12	[64]
Burkholderia kirkii	Vertical	[63]	4	12	[64]
Burkholderia pumila	Vertical	[63]	3.68	12	[64]
Burkholderia schumannianiae	Vertical	[63]	2.36	12	[64]
Burkholderia verschuerenii	Vertical	[63]	6.19	12	[64]
Carsonella ruddii	Vertical	[57]	0.16	120	[57,65]
chemoautotrophic symbiont of *Codakia*	Horizontal	[51]	-	-	-
Coriobacterium glomerans	Vertical	[13]	2.12	-	-
Coxiella	Vertical	[14]	0.66	-	-
Endoriftia persephone	Horizontal	[66]	3.2	-	[66]
Erwinia	Horizontal	[15]	-	-	-
Erwinia dacicola	Vertical	[16]	-	-	-
Frankia	Horizontal	-	7.5	-	-
Gilliamella apicola	Vertical	[67]	2.24	-	-
Glomeribacter gigasporarum	Vertical	[18]	1.73	-	-
Gordonibacter	Vertical	[13]	-	-	-
Hamiltonella defensa	Vertical	[19]	1.84		-
Hodgkinia cicadinicola	Vertical	[68]	0.15	-	-
Ishikawaella capsulata	Vertical	[57]	0.75	-	-

Mesorhizobium loti	Horizontal	-	7.33	62	[61]
Moranella endobia	Vertical	[68]	0.54	-	-
Nardonella	Vertical	[22]	-	-	-
symbiont of *Plautia stali*	Vertical	[23]	-	-	-
Regiella insecticola	Vertical	[19]	2.04		-
Rhodococcus rhodnii	Vertical	[69]	8.4	-	-
Rickettsia	Vertical	-	-	-	-
Riesia pediculicola	Vertical	[53]	0.58	-	-
Rosenkranzia clausaccus	Vertical	[70]	0.94	-	-
Ruthia magnifica	Vertical	[66]	1.2	-	[71]
Serratia symbiotica	Vertical	[19]	3.19	-	-
Sinorhizobium meliloti	Horizontal	-	7	62	[61]
Snodgrassella alvi	Vertical	[72]	2.33	-	-
Spirochaetes bacteria	Vertical	[35]	-	-	-
Streptomyces philanthii	Vertical	[73]	-	68	[73]
Sulcia muelleri	Vertical	[57]	0.21	260	[58]
Tremblaya princeps	Vertical	[68]	0.14	100-200 (150)	[65]
Uzinura diaspidicola	Vertical	[57]	0.26	100	[74]
Verminephrobacter	Vertical	[75]	5.6	62-136 (99)	[75]
Vesicomyosocius okutanii	Vertical	[66]	1	-	[76]
Wigglesworthia glossinidius	Vertical	[57]	0.71	40	[77]
Wolbachia	Vertical	-	-	-	-
Zinderia insecticola	Vertical	[68]	0.21	-	-

Supplementary References

1. Salem, H., Kreutzer, E. & Sudakaran, S. Actinobacteria as essential symbionts in firebugs and cotton stainers (Hemiptera, Pyrrhocoridae) *Environmental Microbiology* **15** 1956-1968 (2013).
2. Kapulnik, Y., Sarig, S., Nur, I. & Okon, Y. Yield Increases in Summer Cereal Crops in Israeli Fields Inoculated with Azospirillum. *Experimental Agriculture* **17,** 179–187 (1981).
3. Rao, R., Nayak, D. N. & Charyulu, P. Yield response of rice to root inoculation with Azospirillum. *Journal of Agricultural Science* **100,** 689–691 (1983).
4. Kapulnik, Y., Okon, Y. & Henis, Y. Yield response of spring wheat cultivars (Triticum aestivum and T. turgidum) to inoculation with Azospirillum brasilense under field conditions. *Biology and Fertility of Soils* **4,** 27–35 (1987).
5. Zientz, E., Beyaert, I., Gross, R. & Feldhaar, H. Relevance of the endosymbiosis of Blochmannia floridanus and carpenter ants at different stages of the life cycle of the host. *Applied and Environmental Microbiology* **72,** 6027–6033 (2006).
6. Feldhaar, H. *et al.* Nutritional upgrading for omnivorous carpenter ants by the endosymbiont Blochmannia. *BMC Biol.* **5,** (2007).
7. Hafeez, F., S, H., T, A. & K, M. Competition between effective and less effective strains of Bradyrhizobium spp. for nodulation on Vigna radiata. *Biology and Fertility of Soils* **33,** 382–386 (2001).
8. Abbasi, M. K., Majeed, A., Sadiq, A. & Khan, S. R. Application of Bradyrhizobium japonicum and Phosphorus Fertilization Improved Growth, Yield and Nodulation of Soybean in the Sub-humid Hilly Region of Azad Jammu and Kashmir, Pakistan. *Plant Prod. Sci.* **11,** 368–376 (2008).
9. Delić, D. & Stajković-Srbinović, O. Delic et al. Effect of bradyrhizobial inoculation on growth and seed yield of mungbean in Fluvisol and Humofluvisol. *African Journal of Microbiology Research* **5,** 3946–3957 (2011).
10. Douglas, A. E. Reproductive failure and the free amino acid pools in pea aphids (Acyrthosiphon pisum) lacking symbiotic bacteria. *Journal of Insect Physiology* **42,** 247–255 (1996).
11. Caillaud, C. M. & Rahbe, Y. Aposymbiosis in a cereal aphid: reproductive failure and influence on plant utilization. *Ecol Entomol* **24,** 111–114 (1999).
12. Kikuchi, Y., Hosokawa, T. & Fukatsu, T. Insect-microbe mutualism without vertical transmission: a stinkbug acquires a beneficial gut symbiont from the environment every generation. *Applied and Environmental Microbiology* **73,** 4308–4316 (2007).
13. Salem, H. *et al.* Vitamin supplementation by gut symbionts ensures metabolic homeostasis in an insect host. *Proceedings of the Royal Society B: Biological Sciences* **281,** (2014).
14. Zhong, J., Jasinskas, A. & Barbour, A. G. Antibiotic Treatment of the Tick Vector Amblyomma americanum Reduced Reproductive Fitness. *PLoS ONE* **2,** 1–7 (2007).
15. de Vries, E. J., Jacobs, G., Sabelis, M. W., Menken, S. B. J. & Breeuwer, J. A. J. Diet-Dependent Effects of Gut Bacteria on Their Insect Host: The Symbiosis of Erwinia sp. and Western Flower Thrips. *Proceedings of the Royal Society B: Biological Sciences* **271,** 2171–2178 (2004).
16. Ben-Yosef, M., Aharon, Y., Jurkevitch, E. & Yuval, B. Give us the tools and we will do the job: symbiotic bacteria affect olive fly fitness in a diet-dependent fashion. *Proceedings of the Royal Society B: Biological Sciences* **277,** 1545–1552 (2010).
17. Oliveira, R. S., Castro, P. M. L., Dodd, J. C. & Vosátka, M. Synergistic effect of Glomus intraradices and Frankia spp. on the growth and stress recovery of Alnus

glutinosa in an alkaline anthropogenic sediment. *Chemosphere* **60,** 1462–1470 (2005).
18. Lumini, E. *et al.* Presymbiotic growth and sporal morphology are affected in the arbuscular mycorrhizal fungus Gigaspora margarita cured of its endobacteria. *Cell. Microbiol.* **9,** 1716–1729 (2007).
19. Russell, J. A. & Moran, N. A. Costs and benefits of symbiont infection in aphids: variation among symbionts and across temperatures. *Proceedings of the Royal Society B: Biological Sciences* **273,** 603–610 (2006).
20. Castañeda, L. E. & Sandrock, C. Variation and covariation of life history traits in aphids are related to infection with the facultative bacterial endosymbiont Hamiltonella defensa. *Biological Journal of the Linnean Society* **100,** 237–247 (2010).
21. Łukasik, P., Dawid, M. A., Ferrari, J. & Godfray, H. C. J. The diversity and fitness effects of infection with facultative endosymbionts in the grain aphid, Sitobion avenae. *Oecologia* 1–12 (2013). doi:10.1007/s00442-013-2660-5
22. Kuriwada, T., Hosokawa, T., Kumano, N. & Shiromoto, K. Biological Role of Nardonella Endosymbiont in Its Weevil Host. *PLoS ONE* **5,** 1–7 (2010).
23. Hosokawa, T., Ishii, Y., Nikoh, N., Fujie, M. & Satoh, N. Obligate bacterial mutualists evolving from environmental bacteria in natural insect populations. *Nature Microbiology.* **1** (2016).
24. Leonardo, T. E. Removal of a specialization-associated symbiont does not affect aphid fitness. *Ecology Letters* **7,** 461–468 (2004).
25. Vorburger, C., Gehrer, L. & Rodriguez, P. A strain of the bacterial symbiont Regiella insecticola protects aphids against parasitoids. *Biology Letters* **6,** 109–111 (2010).
26. Schaub, G. A. & Eichler, S. The effects of aposymbiosis and of an infection with Blastocrithidia triatomae (Trypanosomatidae) on the tracheal system of the reduviid bugs Rhodnius prolixus and Triatoma infestans. *Journal of Insect Physiology* **44,** 131–140 (1998).
27. Chen, D.-Q., Montllor, C. B. & Purcell, A. H. Fitness effects of two facultative endosymbiotic bacteria on the pea aphid, Acyrthosiphon pisum, and the blue alfalfa aphid, A. kondoi. *Entomologia Experimentalis et Applicata* **95,** 315–323 (2000).
28. Montllor, C. B., Maxmen, A. & Purcell, A. H. Facultative bacterial endosymbionts benefit pea aphids Acyrthosiphon pisum under heat stress. *Ecol Entomol* **27,** 189–195 (2002).
29. Sakurai, M., Koga, R., Tsuchida, T., Meng, X.-Y. & Fukatsu, T. Rickettsia Symbiont in the Pea Aphid Acyrthosiphon pisum: Novel Cellular Tropism, Effect on Host Fitness, and Interaction with the Essential Symbiont Buchnera. *Applied and Environmental Microbiology* **71,** 4069–4075 (2005).
30. Chiel, E. *et al.* Assessments of Fitness Effects by the Facultative Symbiont Rickettsia in the Sweetpotato Whitefly (Hemiptera: Aleyrodidae). *Ecology and Population Biology* **102,** 413–418 (2009).
31. Himler, A. G. *et al.* Rapid spread of a bacterial symbiont in an invasive whitefly is driven by fitness benefits and female bias. *Science* **332,** 254–256 (2011).
32. Koga, R., Tsuchida, T. & Fukatsu, T. Changing partners in an obligate symbiosis: a facultative endosymbiont can compensate for loss of the essential endosymbiont Buchnera in an aphid. *Proceedings of the Royal Society B: Biological Sciences* **270,** 2543–2550 (2003).
33. Koga, R., Tsuchida, T., Sakurai, M. & Fukatsu, T. Selective elimination of aphid endosymbionts: effects of antibiotic dose and host genotype, and fitness consequences. *Fems Microbiol Ecol* **60,** 229–239 (2007).
34. Heath, K. D. & Lau, J. A. Herbivores alter the fitness benefits of a plant–rhizobium mutualism. *Acta Oecologica* **37,** 87–92 (2011).

35. Eutick, M. L., Veivers, P., O'Brien, R. W. & Slaytor, M. Dependence of the higher termite, Nasutitermes exitiosus and the lower termite, Coptotermes lacteus on their gut flora. *Journal of Insect Physiology* **24,** 363–368 (1978).
36. Fukatsu, T., Tsuchida, T., Nikoh, N. & Koga, R. Spiroplasma Symbiont of the Pea Aphid, Acyrthosiphon pisum (Insecta: Homoptera). *Applied and Environmental Microbiology* (2001).
37. Xie, J., Vilchez, I. & Mateos, M. Spiroplasma Bacteria Enhance Survival of Drosophila hydei Attacked by the Parasitic Wasp Leptopilina heterotoma. *PLoS ONE* **5,** 1–7 (2010).
38. Kaltenpoth, M., Gottler, W., Herzner, G. & Strohm, E. Symbiotic bacteria protect wasp larvae from fungal infestation. *Current Biology* **15,** 475–479 (2005).
39. Lund, M. B., Holmstrup, M., Lomstein, B. A., Damgaard, C. & Schramm, A. Beneficial Effect of Verminephrobacter Nephridial Symbionts on the Fitness of the Earthworm Aporrectodea tuberculata. *Applied and Environmental Microbiology* **76,** 4738–4743 (2010).
40. Pais, R., Lohs, C., Wu, Y., Wang, J. & Aksoy, S. The obligate mutualist Wigglesworthia glossinidia influences reproduction, digestion, and immunity processes of its host, the tsetse fly. *Applied and Environmental Microbiology* **74,** 5965–5974 (2008).
41. Hoffmann, A. A., Clancy, D. J. & Merton, E. Cytoplasmic incompatibility in Australian populations of Drosophila melanogaster. *Genetics* **136,** 993–999 (1994).
42. Fleury, F., Vavre, F., Ris, N. & Fouillet, P. Physiological cost induced by the maternally-transmitted endosymbiont Wolbachia in the Drosophila parasitoid Leptopilina heterotoma. *Parasitology* **121,** 493–500 (2000).
43. Dedeine, F. *et al.* Removing symbiotic Wolbachia bacteria specifically inhibits oogenesis in a parasitic wasp. *Proceedings of the Royal Society B: Biological Sciences* **98,** 6247–6252 (2001).
44. Rao, R. & Weil, G. J. In vitro Effects of Antibiotics on Brugia malayi Worm Survival and Reproduction. *The Journal of Parasitology* **88,** 605–611 (2002).
45. Vala, F., Van Opijnen, T., Breeuwer, J. A. J. & Sabelis, M. W. Genetic Conflicts over Sex Ratio: Mite- Endosymbiont Interactions. *The American Naturalist* **161,** 254–266 (2003).
46. Dedeine, F., Boulétreau, M. & Vavre, F. Wolbachia requirement for oogenesis: occurrence within the genus Asobara (Hymenoptera, Braconidae) and evidence for intraspecific variation in A. tabida. *Heredity* **95,** 394–400 (2005).
47. Fytrou, A., Schofield, P. G., Kraaijeveld, A. R. & Hubbard, S. F. Wolbachia Infection Suppresses Both Host Defence and Parasitoid Counter-Defence. *Proceedings of the Royal Society B: Biological Sciences* **273,** 791–796 (2006).
48. Brownlie, J. C., Cass, B. N., Riegler, M. & Witsenburg, J. J. Evidence for Metabolic Provisioning by a Common Invertebrate Endosymbiont, Wolbachia pipientis, during Periods of Nutritional Stress. *PLoS Pathogens* **5,** 1–5 (2009).
49. Ikeya, T., Broughton, S., Alic, N., Grandison, R. & Partridge, L. The Endosymbiont Wolbachia Increases Insulin/IGF-Like Signalling in Drosophila. *Proceedings: Biological Sciences* **276,** 3799–3807 (2009).
50. Hosokawa, T., Koga, R., Kikuchi, Y., Meng, X.-Y. & Fukatsu, T. Wolbachia as a bacteriocyte-associated nutritional mutualist. *Proceedings of the National Academy of Sciences of the United States of America* **107,** 769–774 (2010).
51. Gros, O., Frenkiel, L. & Mouëza, M. Gill filament differentiation and experimental colonization by symbiotic bacteria in aposymbiotic juveniles of Codakia orbicularis (Bivalvia: Lucinidae). *Invertebrate Reproduction & Development* **34,** 219–231 (1998).

52. Salem, H., Florez, L., Gerardo, N. & Kaltenpoth, M. An out-of-body experience: the extracellular dimension for the transmission of mutualistic bacteria in insects. *Proceedings of the Royal Society B: Biological Sciences* **282,** (2015).
53. Bright, M. & Bulgheresi, S. A complex journey: transmission of microbial symbionts. *Nat Rev Microbiol* **8,** 218–230 (2010).
54. Ran, L. *et al.* Genome erosion in a nitrogen-fixing vertically transmitted endosymbiotic multicellular cyanobacterium. *PLoS ONE* **5,** e11486 (2010).
55. Peters, G. A. & Mayne, B. C. The Azolla, Anabaena azollae Relationship. *Plant Physiology* (1974).
56. Hongoh, Y., Sharma, V. K., Prakash, T., Noda, S. & Toh, H. Genome of an Endosymbiont Coupling N2 Fixation to Cellulolysis Within Protist Cells in Termite Gut. *Science* (2008).
57. Moran, N. A., McCutcheon, J. P. & Nakabachi, A. Genomics and Evolution of Heritable Bacterial Symbionts. *Annual Review of Genetics* **42,** 165–190 (2008).
58. Moran, N. A., Russell, J. A., Koga, R. & Fukatsu, T. Evolutionary Relationships of Three New Species of Enterobacteriaceae Living as Symbionts of Aphids and Other Insects. *Applied and Environmental Microbiology* **71,** 3302–3310 (2005).
59. Lo, N., Bandi, C., Watanabe, H., Nalepa, C. & Beninati, T. Evidence for cocladogenesis between diverse dictyopteran lineages and their intracellular endosymbionts. *Mol. Biol. Evol.* **20** 907-913 (2003).
60. Sauer, C., Stackebrandt, E., Gadau, J., Holldobler, B. & Gross, R. Systematic relationships and cospeciation of bacterial endosymbionts and their carpenter ant host species: proposal of the new taxon Candidatus Blochmannia gen. nov. *Int. J. Syst. Evol. Microbiol.* **50,** 1877–1886 (2000).
61. Werner, G. D. A., Cornwell, W. K., Sprent, J. I., Kattge, J. & Kiers, E. T. A single evolutionary innovation drives the deep evolution of symbiotic N2-fixation in angiosperms. *Nat Communications* **5** (2014).
62. Douglas, A. E. Host benefit and the evolution of specialization in symbiosis. *Heredity* **81,** 599–603 (1998).
63. Lemaire, B., Janssens, S., Smets, E. & Dessein, S. Endosymbiont Transmission Mode in Bacterial Leaf Nodulation as Revealed by a Population Genetic Study of Psychotria leptophylla. *Applied and Environmental Microbiology* **78,** 284–287 (2012).
64. Lemaire, B., Vandamme, P., Merckx, V., Smets, E. & Dessein, S. Bacterial Leaf Symbiosis in Angiosperms: Host Specificity without Co-Speciation. *PLoS ONE* **6,** 1–10 (2011).
65. Baumann, P. Biology bacteriocyte-associated endosymbionts of plant sap-sucking insects. *Annu. Rev. Microbiol.* **59,** 155–189 (2005).
66. Kleiner, M., Petersen, J. M. & Dubilier, N. Convergent and divergent evolution of metabolism in sulfur-oxidizing symbionts and the role of horizontal gene transfer. *Current Opinion in Microbiology* **15,** 621–631 (2012).
67. Kwong, W. K. & Moran, N. A. Cultivation and characterization of the gut symbionts of honey bees and bumble bees: description of Snodgrassella alvi gen. nov., sp. nov., a member of the family Neisseriaceae of the Betaproteobacteria, and Gilliamella apicola gen. nov., sp. nov., a member of Orbaceae fam. nov., Orbales ord. nov., a sister taxon to the order 'Enterobacteriales' of the Gammaproteobacteria. *Int. J. Syst. Evol. Microbiol.* **63,** 2008–2018 (2013).
68. McCutcheon, J. P. & Moran, N. A. Extreme genome reduction in symbiotic bacteria. *Nat Rev Microbiol* **10,** 13–26 (2011).
69. Brecher, G. & Wigglesworth, V. B. The transmission of Actinomyces rhodnii Erikson in Rhodnius prolixus (Hemiptera) and its influence on the growth of the host.

Parasitology **35,** 220 (1943).
70. Kikuchi, Y. *et al.* Host-symbiont co-speciation and reductive genome evolution in gut symbiotic bacteria of acanthosomatid stinkbugs. *BMC Biol.* **7,** 2 (2009).
71. Newton, I. L. G. *et al.* The Calyptogena magnifica Chemoautotrophic Symbiont Genome. *Science* **315,** 998–1000 (2007).
72. Koch, H. & Schmid-Hempel, P. Socially transmitted gut microbiota protect bumble bees against an intestinal parasite. *Proceedings of the National Academy of Sciences of the United States of America* **108,** 19288–19292 (2011).
73. Kaltenpoth, M. & Engl, T. Defensive microbial symbionts in Hymenoptera. *Funct Ecology* **28,** 315–327 (2014).
74. Gruwell, M. E., Morse, G. E. & Normark, B. B. Phylogenetic congruence of armored scale insects (Hemiptera : Diaspididae) and their primary endosymbionts from the phylum Bacteroidetes. *Mol. Phylogenet. Evol.* **44,** 267–280 (2007).
75. Lund, M. B., Kjeldsen, K. U. & Schramm, A. The earthworm - Verminephrobacter symbiosis: an emerging experimental system to study extracellular symbiosis. *Front Microbiol* **5,** (2014).
76. Kuwahara, H. *et al.* Reduced Genome of the Thioautotrophic Intracellular Symbiont in a Deep-Sea Clam, Calyptogena okutanii. *Current Biology* **17,** 881–886 (2007).
77. Chen, X., Li, S. & Aksoy, S. Concordant Evolution of a Symbiont with Its Host Insect Species: Molecular Phylogeny of Genus Glossina and Its Bacteriome-Associated Endosymbiont, Wigglesworthia glossinidia. *J Mol Evol* **48,** 49–58 (1999).
